# When cyclopropenes meet gold catalysts

**DOI:** 10.3762/bjoc.7.82

**Published:** 2011-05-30

**Authors:** Frédéric Miege, Christophe Meyer, Janine Cossy

**Affiliations:** 1 Laboratoire de Chimie Organique, ESPCI ParisTech, CNRS (UMR 7084), 10 rue Vauquelin 75231 Paris Cedex 05, France

**Keywords:** cyclopropenes, gold carbenes, gold catalysis, gold-stabilized allylic cations, ring-opening

## Abstract

Cyclopropenes as substrates entered the field of gold catalysis in 2008 and have proven to be valuable partners in a variety of gold-catalyzed reactions. The different contributions in this growing research area are summarized in this review.

## Review

### Introduction

Homogeneous gold catalysis has become a particularly active research area over the last decade. The ability of gold catalysts to act as potent carbophilic Lewis acids and hence to chemoselectively activate π bonds towards nucleophilic attack is now well-established and has found many impressive applications for the formation of C–C or C–heteroatom bonds [1–14]. Whereas alkynes, alkenes and allenes have been widely used as substrates or partners in gold-catalyzed reactions, it was only rather recently, in 2008, that cyclopropenes entered the field of gold catalysis despite their well-known high and versatile reactivity in transition metal-catalyzed reactions [15].

**Scheme 1:**
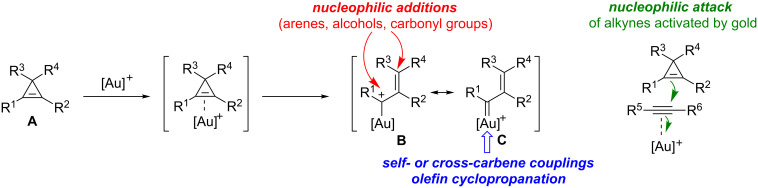
General reactivity of cyclopropenes in the presence of gold catalysts.

As has been observed with other transition metals, the reactivity of cyclopropenes **A** in gold-catalyzed reactions is essentially (but not exclusively) related to their ability to act as ligands for π-acidic gold complexes, and hence, to undergo subsequent ring-opening to produce an organogold species that can be viewed as a hybrid between a gold-stabilized allylic carbocation **B** and a gold carbene **C**. The organogold carbenoid species generated by the ring-opening of cyclopropenes can participate in a variety of reaction types such as nucleophilic addition with, e.g., alcohols, arenes or carbonyl groups, undergo self- or cross-carbene couplings and bring about the cyclopropanation of olefins. The first of these reaction types is often considered to be representative of cationic intermediates whereas the other two are best ascribed to carbene-like reactivity, although this distinction is artificial. Alternatively, cyclopropenes can also behave as nucleophiles and attack other functional groups that are more readily activated by gold complexes, such as alkynes (Scheme 1) [16–26].

This review illustrates the different aspects of the reactivity of cyclopropenes in the presence of gold catalysts and covers the contributions in this field up to February 2011.

Besides their implication in several gold-catalyzed reactions, cyclopropenes have also served as substrates in order to gain insight into the gold-carbon order in the so-called organogold carbenoids. In the broad repertoire of gold-catalyzed organic transformations, gold-stabilized carbocations or, more often gold carbenes, can be found as intermediates in proposed mechanistic pathways, but the true nature of the organogold species had been a matter of debate [27].

### Structural considerations: Gold-stabilized carbocations or gold carbenes?

**Scheme 2:**
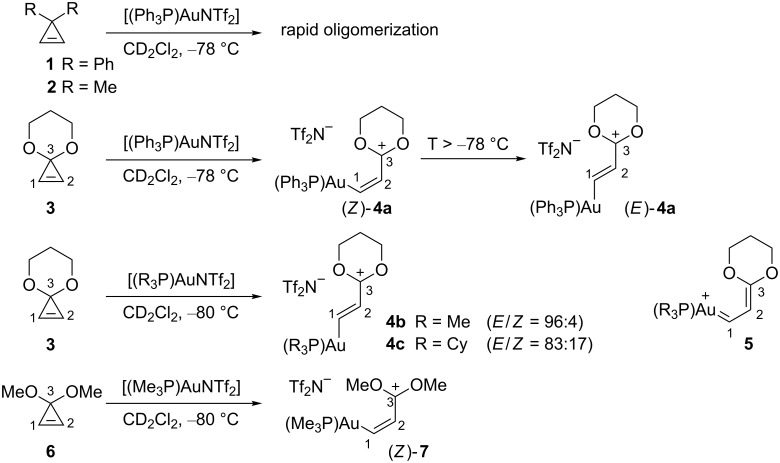
Cationic organogold species generated from cyclopropenone acetals.

In 2008, Fürstner et al. took advantage of the ring-opening of 3,3-disubstituted cyclopropenes to generate organogold species and characterize them by NMR spectroscopy [16]. Whereas, 3,3-diphenylcyclopropene (**1**) or 3,3-dimethylcyclopropene (**2**) did not generate a defined organogold species upon treatment with Gagosz’s complex [(Ph_3_P)AuNTf_2_] [28] (CD_2_Cl_2_, −78 °C) due to rapid oligomerization, the cyclopropenone acetal **3** gave an organogold species whose NMR spectroscopic data corresponds to the carbocationic structure (*Z*)-**4a**. Upon raising the temperature, organogold (*Z*)-**4a** was found to isomerize into its geometric isomer (*E*)-**4a**. Switching to more electron-donating phosphine ligands such as PMe_3_ or PCy_3_, also led to the organogold species **4b** and **4c**, respectively, possessing a dioxacarbenium structure, with the predominance of the *E* geometric isomers already at −80 °C. The observed data point towards a high degree of double bond character for the C1–C2 bond, and not the C2–C3 bond, in the organogold species generated by ring-opening of cyclopropenone acetal **3**, with a marginal contribution of the carbene form **5**. The magnitude of the rotational barrier around the C2–C3 bond for **4a** (<30 kJ·mol^−1^) was in agreement with this result. In the case of the less stable organogold species (*Z*)-**7**, generated from 3,3-dimethoxycyclopropene (**6**) using [(Me_3_P)AuNTf_2_], the broadening of the NMR signals indicated a more restricted rotation around the C2–C3 bond at −80 °C, but the rotation barrier estimated to be 46 ± 1 kJ·mol^–1^ was still comparable in magnitude to rotation around a sterically hindered σ bond (such as in hexachloroethane) (Scheme 2) [16].

These experiments appeared to be useful for the determination of the cationic or carbenic nature of organogold intermediates, but the presence of the two oxygen atoms in cyclopropenone acetals unavoidably led to more favorable cationic forms and hence cannot provide a general answer.

**Scheme 3:**
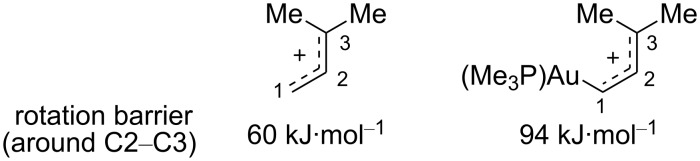
Rotation barriers around the C2–C3 bond (M06 DFT calculations).

Using the M06 functional of DFT, Toste et al. calculated rotational barriers for (*Z*)-**4a** and (*Z*)-**7** and the results were found to be in agreement with those previously obtained experimentally by Fürstner et al. Thus, with this validated computational method, the barriers to bond rotation in (metal free) 3,3-disubstituted allyl cations, and in the corresponding (Me_3_P)Au-substituted organogold species, were calculated. Unlike in the case of the allylic cation bearing an acetal moiety at C3, incorporation of the gold center at C1 in the 3,3-dimethyl substituted allylic cation raised the rotation barrier considerably to 94 kJ·mol^−1^, and hence the latter species should be regarded more as a gold carbene (Scheme 3) [17].

**Scheme 4:**
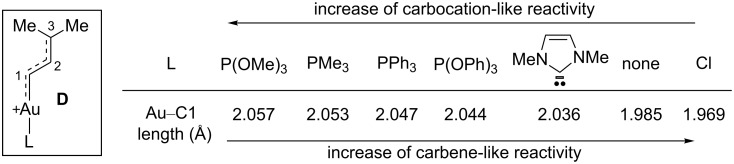
Au–C1 bond length in organogold species of type **D**.

The bond distances and natural atomic charges were calculated for a series of 3,3-disubstituted allylic cations, bearing an acetal, two methyl or two carbomethoxy groups, as well as for their (trimethylphosphine)gold-substituted counterparts. The results indicate that a secondary gold-substituted carbocation (at C1) is as stable as a tertiary dimethyl-substituted carbocation (at C3) and that the magnitude of stabilization from the gold moiety increases with increasing electrophilicity of the allylic cation. Toste et al. investigated the effect of the ligand on the structure of gold-substituted 3,3-dimethyl allyl cations of type **D**. Increasing *trans* σ-donation from the ligand and strongly π-acidic ligands such as phosphites (decreasing back π-donation from gold to C1) led to a longer C1–Au bond and hence a more carbocation-like character for the organogold species. By contrast, those ligands that increase gold-to-C1 back π-donation or decrease C1-to-gold σ-donation will induce a shorter C1–Au bond and a carbene-like reactivity (Scheme 4) [17].

These studies highlighted the tremendous influence of the substitution pattern and the ancillary ligand on the nature of bonding in cationic gold-stabilized intermediates. Interestingly, the organogold species investigated in these computational studies are precisely those that can be generated by the ring-opening of cyclopropenes in the presence of gold complexes. Indeed, as it will be illustrated later in this review, these structural effects were found to have important consequences in terms of reactivity in the case of intermolecular olefin cyclopropanation promoted by gold carbenes generated from cyclopropenes.

In fact, the first reports on gold-catalyzed reactions involving cyclopropenes appeared in the literature before these structural investigations were carried out. In the following presentation of the different chemical transformations involving cyclopropenes, either one of the two forms (i.e., an allylic gold cation or carbene) will be drawn in the mechanistic pathway. In general, little information is available on the modulation and tuning of the reactivity by the choice of the gold ligand.

### Nucleophilic addition to gold-stabilized allylic cations generated from cyclopropenes

#### Intermolecular addition of oxygen nucleophiles

**Scheme 5:**
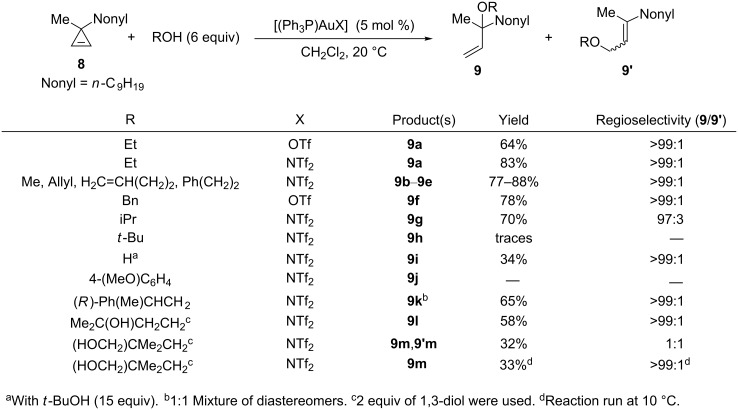
Gold-catalyzed addition of alcohols or water to cyclopropene **8**.

In 2008, Lee et al. reported several gold-catalyzed reactions involving cyclopropenes among which the addition of alcohols to 3-methyl-3-nonylcyclopropene (**8**) was investigated in detail [18]. A variety of primary alcohols reacted with cyclopropene **8** in the presence of either in situ generated [(Ph_3_P)AuOTf] or [(Ph_3_P)AuNTf_2_] (5 mol %) to afford the corresponding *tert*-allylic ethers **9a**–**9f** with very high regioselectivity (>99%). Other catalysts such as AuCl_3_ or Rh_2_(OAc)_4_ provided mixtures of compounds containing traces of allylic ethers **9** and **9’** and mostly oxidation products (vide infra, enals **16** and **17**). AgOTf was less efficient and led to an incomplete conversion whereas no reaction took place with TfOH. Gagosz’s catalyst was in general more efficient than [(Ph_3_P)AuOTf] and also allowed iPrOH to be used as a nucleophile to provide ether **9g** (70%), however, tertiary alcohols did not react under these conditions. Water in the presence of *t*-BuOH as co-solvent acted as a nucleophile, but the corresponding tertiary alcohol **9i** was isolated in only modest yield (34%) (Scheme 5) [18]. Additional results were subsequently reported by Lee et al. in a full article in 2010 [19]. Due to their lower nucleophilic character compared to alcohols, phenols could not be used. With the optically pure chiral alcohol (*R*)-PhMeCHCH_2_OH as a nucleophile, the reaction was not diastereoselective and led to the tertiary allylic ether **9k** (65%) as a 1:1 mixture of diastereomers. An unprotected primary and tertiary 1,3-diol reacted chemoselectively with the primary alcohol to furnish monoether **9l** (58%). Addition of neopentyl glycol led to a 1:1 mixture of regioisomeric monoethers **9m** and **9’m** in modest yield (32%) due to the competitive formation of oligomeric by-products. The regioselectivity was found to be highly sensitive to temperature since the tertiary monoether **9m** was selectively obtained (**9m**/**9’m** > 99:1) (33%) when the reaction was carried out at 10 °C (Scheme 5) [18–19].

**Scheme 6:**
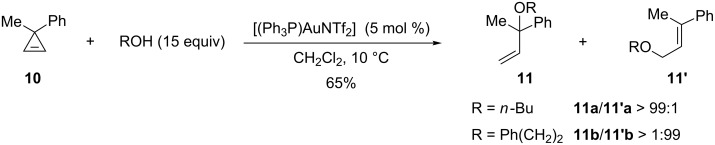
Gold-catalyzed addition of alcohols to cyclopropene **10**.

The reaction was successfully extended to a variety of 3,3-disubstituted cyclopropenes (3-methyl-3-benzylcyclopropene, spiro[2.5]oct-1-ene, 3-benzyl-3-isopropylcyclopropene, 3-*tert*-butyl-3-methylcyclopropene) and the corresponding tertiary allylic ethers were always obtained with high regioselectivities (92:8 to >99:1). However, when 3-methyl-3-phenylcyclopropene (**10**) was used as the substrate the regioselectivity was altered in some cases. With *n*-BuOH as a nucleophile, a 1:1 regioisomeric mixture of allylic ethers **11a** and **11’a** was obtained under the previously used reaction conditions. By lowering the temperature to 10 °C and increasing the quantity of *n*-BuOH (15 equiv), the tertiary allylic ether **11a** (65%) was obtained regioselectively (**11a**/**11’a** > 99:1). Curiously, a complete switch of the regioselectivity took place when phenethyl alcohol was employed as a nucleophile, since in this case the primary allylic ether **11’b** (65%) was obtained (**11b**/**11’b** > 1:99) (Scheme 6) [19].

**Scheme 7:**
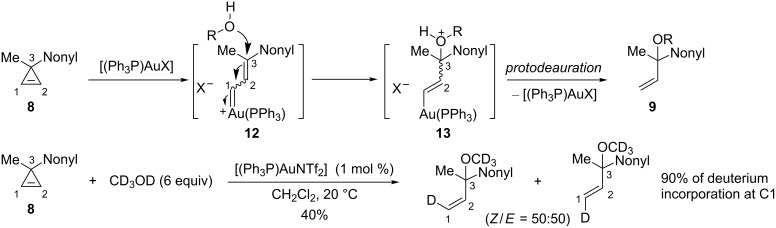
Mechanism of the gold-catalyzed addition of alcohols to cyclopropenes.

The formation of the *tert*-allylic ethers **9** can be explained by the regioselective attack of the alcohol at C3 on the organogold species **12**, generated by electrophilic ring-opening of cyclopropene **8**, followed by protodeauration of the resulting vinyl gold species **13**. Using CD_3_OD as a nucleophile effectively led to 90% deuterium incorporation at C1 and formation of a mixture of geometric isomers (Scheme 7) [18–19].

**Scheme 8:**
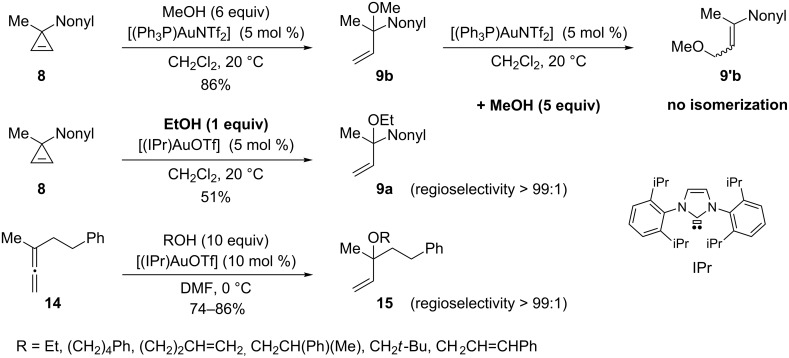
Synthesis of *tert*-allylic ethers from cyclopropenes and allenes.

Interestingly, an excess of the alcohol (6 equiv) was crucial to achieve high regioselectivities. If the quantity of EtOH was reduced (1 equiv) a 2:1 mixture of the corresponding regioisomeric allylic ethers **9a** and **9’a** was obtained, however, the addition of a protic additive [*t*-BuOH (5 equiv)] restored the high regioselectivity (>99:1) [18–19]. Lee and Hadfield demonstrated that the use of an excess of methanol retarded the isomerization of the tertiary allylic ether **9b** into the primary allylic isomer **9’b**, which is also catalyzed by the gold complex [29] (Scheme 8). The isomerization was also found to be catalyst dependent and did not operate in the presence of the NHC–gold complex [(IPr)AuOTf]. Thus, when cyclopropene **8** was treated with a stoichiometric quantity of EtOH in the presence of the latter catalyst (5 mol %), the tertiary allylic ether **9a** was obtained with high regioselectivity (>99:1), but the yield (51%) was not as high as with Gagosz’s catalyst (83%). Lee and Hadfield took advantage of these findings to develop the regioselective addition of alcohols (used in excess) to allenes such as **14** catalyzed by [(IPr)AuOTf] (10 mol %) to produce the *tert*-allylic ethers **15** as the kinetic products (Scheme 8) [29].

**Scheme 9:**
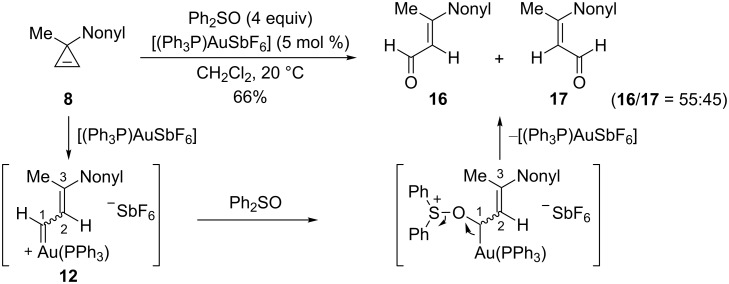
Oxidation of the intermediate gold–carbene with diphenylsulfoxide.

During their studies on the addition of alcohols to cyclopropenes, Lee et al. also reported one example of oxidation of the gold carbene intermediate **12**, resulting from the electrophilic ring-opening of 3-methyl-3-nonylcyclopropene (**8**), with diphenylsulfoxide [30]. The reaction proceeds by nucleophilic attack of diphenylsulfoxide at C1 followed by elimination of diphenylsulfide to afford a 55:45 mixture of the *E* and *Z* enals **16** and **17**, respectively (66%) (Scheme 9) [18–19].

Other examples of nucleophilic attack on organogold species resulting from the ring-opening of cyclopropenes in the presence of gold complexes involve intramolecular Friedel–Crafts reactions and the addition of carbonyl groups.

#### Intramolecular Friedel–Crafts reactions

**Scheme 10:**
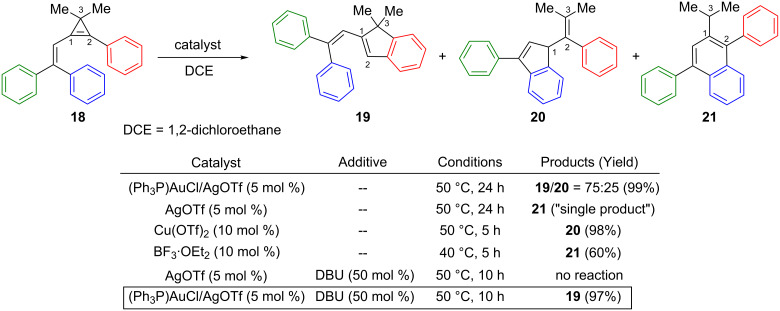
Gold, copper and Lewis acid-catalyzed reactions of cyclopropene **18**.

In the context of their studies on the Lewis acid-catalyzed rearrangement of strained three-membered ring hydrocarbons, such as methylenecyclopropanes and vinylidenecyclopropanes, Shi et al. investigated the behaviour of 1-(2,2-diarylvinyl)-2-phenylcyclopropenes in the presence of gold catalysts [20]. Upon treatment with [(Ph_3_P)AuOTf], vinylcyclopropene **18** was found to produce a mixture of regioisomeric indenes **19** and **20** in a 75:25 ratio (99%). The use of AgOTf alone led to the isomeric substituted naphthalene **21** as the sole product. Shi et al. had previously demonstrated that indene **20** and naphthalene **21** could be selectively formed using Cu(OTf)_2_ and BF_3_·OEt_2_ as catalysts, respectively, thereby highlighting the complementarities of the different electrophilic activators [31]. Since AgOTf and BF_3_·OEt_2_ led to the same naphthalene product **21**, the authors suspected that traces of the Brønsted acid (HOTf) present in the silver salt may be the actual catalyst and may also modify the regioselectivity observed in the gold-catalyzed reaction. Thus, several basic additives were screened and it was found that DBU not only inhibited the isomerization of vinylcyclopropene **18** in the presence of AgOTf, but also led to a completely regioselective gold-catalyzed process to afford indene **19** as the sole reaction product (97%) (Scheme 10) [20].

**Scheme 11:**
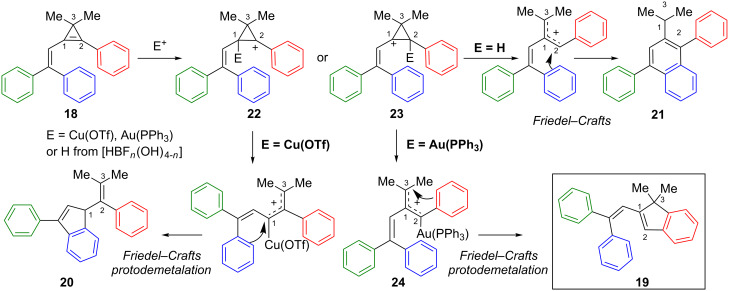
Mechanism of the Lewis acid-catalyzed reactions of cyclopropene **18**.

Upon electrophilic activation, vinylcyclopropene **18** can give rise to two regioisomeric cyclopropyl cations **22** and **23**. It is worth noting that consideration of these two cation species is only helpful to understand the observed regioselectivities, though they may not be actually involved as intermediates during the ring-opening of cyclopropenes in the presence of electrophilic transition metal complexes. Shi et al. initially suggested that the formation of **22** preferentially occurs with a rather bulky electrophile such as Cu(OTf)_2_ to avoid repulsion with the aryl group at C2. Conversely, a Brønsted acid (generated by reaction of BF_3_·OEt_2_ with traces of water) should favour the formation of the more stable cyclopropyl cation **23**. Afterwards, ring-opening and intramolecular Friedel–Crafts reactions should enable the formation of indene **20** or naphthalene **21**. With the gold catalyst, the formation of indene **19** indicated that electrophilic activation of the cyclopropene **18** also occurred at C2 to afford, after ring-opening, the gold-stabilized allylic cation **24**. However, in contrast to the acid-catalyzed reaction, subsequent intramolecular Friedel–Crafts cyclization occurred by nucleophilic attack by the phenyl group (at C2) on the organogold species at C3, followed by protodeauration (Scheme 11) [20,31].

**Scheme 12:**
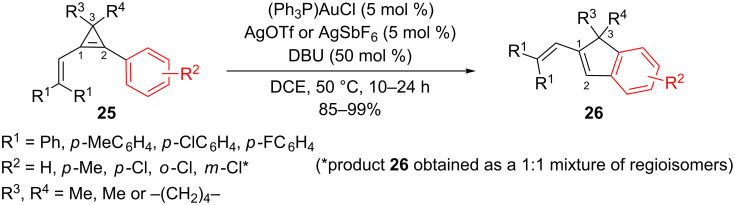
Gold-catalyzed rearrangement of vinylcyclopropenes **25**.

The reaction was generalized with a series of 3,3-disubstituted-1-(2,2-diarylvinyl)-2-arylcyclopropenes of general formula **25**. The catalyst [(Ph_3_P)AuSbF_6_] was found to provide better results than [(Ph_3_P)AuOTf] for substrates having electron-withdrawing substituents on the benzene rings. The corresponding indenes **26** were obtained in good to excellent yields (85–99%) under the previously optimized conditions (Scheme 12) [20].

In the absence of substituents at C3 (R^3^ = R^4^ = H), or when a single substituent was attached to this carbon (R^3^ = Me, R^4^ = H), the reaction led to a complex mixture of products. The authors attributed these results to the formation of less stable carbocations at C3 (primary or secondary, respectively).

**Scheme 13:**
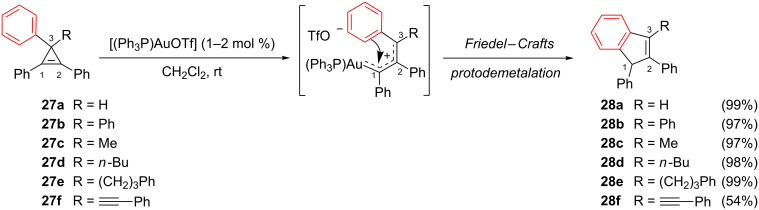
Gold-catalyzed rearrangement of cyclopropenes **27** to indenes **28**.

Other examples of gold-catalyzed isomerization of cyclopropenes that involve a Friedel–Crafts cyclization have been reported. In 2009, Wang et al. demonstrated that [(Ph_3_P)AuOTf] could smoothly catalyze the isomerization of a variety of 3-substituted 1,2,3-triphenylcyclopropenes **27** into 3-substituted 1,2-diphenyl-1*H*-indenes **28** [21]. The rearrangement occurred rapidly (20–40 min) for substrates **27a**–**27e** and indenes **28a**–**28e** were obtained in excellent yields (97–99%). A phenylethynyl group could be present at C3, but the rearrangement of substrate **27f** proceeded slowly (rt, 6 h) and gave indene **28f** in only a moderate yield (54%) together with an unknown by-product, presumably because the alkyne competes with the cyclopropene for coordination to the gold catalyst (Scheme 13) [21,26].

The rearrangement of **27a** to **28a** (95%) had been previously reported by Müller et al. using rhodium(II) perfluorobutyrate as a catalyst (1 mol %, C_6_H_6_, reflux, 48 h) [32], whereas Padwa et al. showed that the isomerization of **27b** to **28b** was quantitatively catalyzed by AgClO_4_ (2 mol %, C_6_H_6_, rt) [33].

**Scheme 14:**
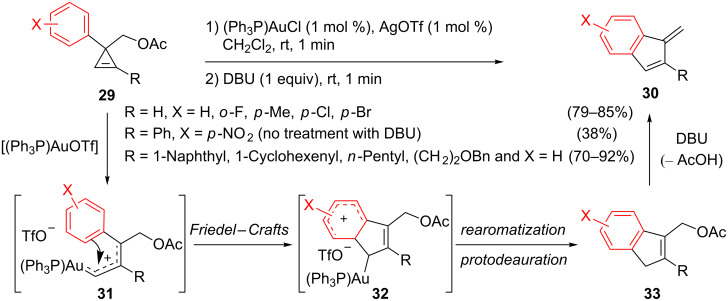
Gold-catalyzed rearrangement of cyclopropenes **29** to indenes **30**.

Wang et al. also examined the behaviour of 3-arylcyclopropenes bearing a protected hydroxymethyl group at C3: only acetates **29** underwent clean conversion to 1-methylene-2-substituted-1*H*-indenes **30** [21]. The yields were improved by the addition of DBU once the rearrangement was complete. For substrates **29** possessing an unsymmetrically substituted endocyclic olefin, it is worth noting that electrophilic activation of the cyclopropene occurred regioselectively to produce the organogold species **31** (formally resulting from the ring-opening of a secondary benzylic cyclopropyl cation). The gold carbene **31** was captured by the aromatic group at C3 via an intramolecular Friedel–Crafts reaction. Subsequent elimination of AcOH from compound **33** then delivered methylene indene **30** (Scheme 14) [21].

Other gold-catalyzed rearrangements of cyclopropenes that proceed through ring-opening and intramolecular Friedel–Crafts cyclization have been studied using 3-aryl-cyclopropene-3-carboxylates. However, for these latter substrates, the carbonyl group can also play the role of a nucleophile and compete with the aryl group.

#### Nucleophilic addition of carbonyl groups in competition with Friedel–Crafts reactions

**Scheme 15:**
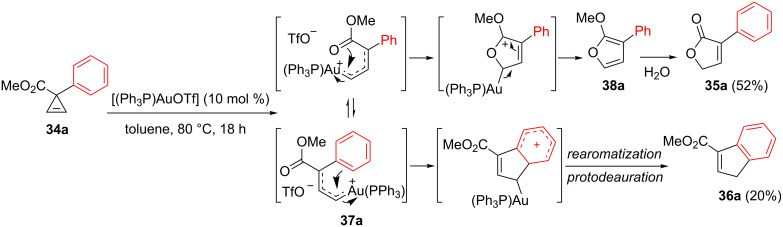
Gold-catalyzed rearrangement of cyclopropenyl ester **34a**.

Besides the gold-catalyzed intermolecular addition of alcohols to cyclopropenes, Lee et al. investigated the behaviour of methyl 3-arylcyclopropen-2-yl carboxylates to ascertain whether the organogold species resulting from the ring-opening in the presence of [(Ph_3_P)AuOTf] (10 mol %) would be trapped in an intramolecular fashion, either by the oxygen atom of the carbonyl group, or by the phenyl group [18]. For cyclopropene **34a** possessing an unsubstituted endocyclic alkene, heating in toluene (80 °C, 18 h) was required and the reaction afforded two products: Furanone **35a** (52%) and indene **36a** (20%). The former compound arose from intramolecular trapping of the intermediate organogold species **37a** by the carbonyl group of the ester at C3, followed by hydrolysis of the resulting α-methoxyfuran **38a**. A similar result was reported by Wang et al. [21]. Indene **36a** is, as previously mentioned, the product resulting from an intramolecular Friedel–Crafts reaction (Scheme 15).

**Scheme 16:**
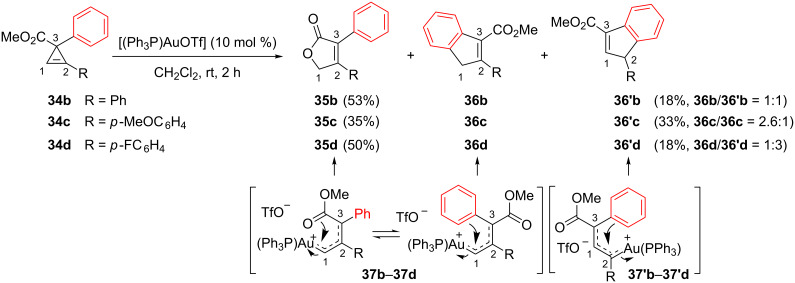
Gold-catalyzed reactions of cyclopropenyl esters **34b**–**34d**.

For unsymmetrical cyclopropenes **34b**–**34d** possessing a trisubstituted endocyclic double bond, the rearrangement took place at rt and invariably led to mixtures of furanones **35b**–**35d**, and mixtures of the inseparable regioisomeric indenes **36b**–**36d** and **36’b**–**36’d**. Electrophilic activation and ring-opening of cyclopropenes **34** favored the formation of the organogold species **37b**–**37d**. Furanones **35b**–**35d** and indenes **36b**–**36d** result from nucleophilic attack on these latter intermediates at C1 by the carbonyl or the phenyl group, respectively. By contrast, the regioisomeric indenes **36’b**–**36’d** would arise from the initial formation of organogold species **37’b**–**37’d** and subsequent Friedel–Crafts cyclization (Scheme 16) [18].

**Scheme 17:**
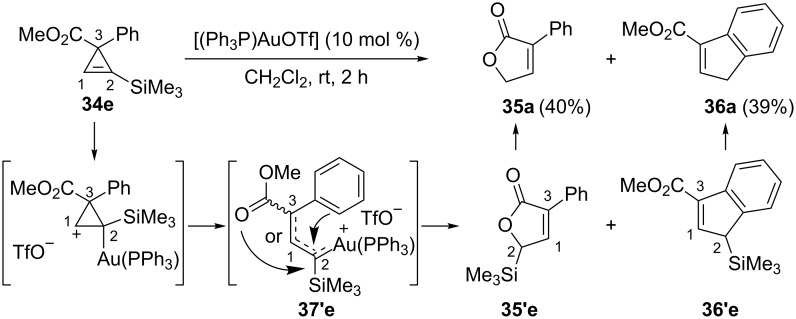
Gold-catalyzed reactions of cyclopropenylsilane **34e**.

Interestingly, the gold-catalyzed rearrangement of cyclopropenylsilane **34e** provided two compounds: Furanone **35a** (40%) and indene **36a** (39%) both devoid of a trimethylsilyl group. Since protodesilylation took place readily, it is likely that the allylic silanes **35’e** and **36’e** were the initially generated products. Their formation could be explained by regioselective electrophilic activation and ring-opening of the cyclopropenylsilane leading to the organogold **37’e** (formally arising from ring-opening of a cyclopropyl cation at the β-position of the trimethylsilyl group). Subsequent nucleophilic attack by the carbonyl and the phenyl would produce **35’e** and **36’e**, though this was not discussed by the authors (Scheme 17) [18].

It is worth noting that for substrates bearing two electron-withdrawing groups at C3 (COMe and CO_2_Me), no gold-catalyzed rearrangement took place under similar conditions. However, such cyclopropenes have been converted to furans in the presence of CuI or PdCl_2_(MeCN)_2_ as catalysts [34].

#### Rearrangement of cyclopropenylmethyl acetates

**Scheme 18:**
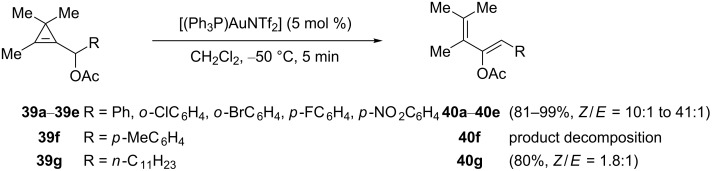
Gold-catalyzed rearrangement of cyclopropenylmethyl acetates.

Propargylic carboxylates have proven to be particularly interesting substrates in gold-catalyzed reactions that have led to the development of useful synthetic processes relying on 1,3- or 1,2-acyloxy migration as the key step, depending on the substitution pattern [35–36]. Due to their high strain and π-electron density, cyclopropenes exhibit reactivity often comparable to that of alkynes in transition metal-catalyzed reactions. Not surprisingly, the reactivity of cyclopropenylmethyl carboxylates in the presence of gold catalysts has been investigated as reported in 2010 by Ariafard, Hyland et al. [22]*.* These authors reported that 2,3,3-trimethyl-cyclopropenylmethyl acetates **39** underwent a gold-catalyzed rearrangement into the corresponding 2-acetoxydienes **40**, and a screening of gold catalysts indicated the superior activity of Gagosz’s complex [(Ph_3_P)AuNTf_2_] in terms of yield and selectivity. Starting from arylcyclopropenylmethyl acetates **39a**–**39e** substituted by a phenyl group or an electron-deficient aromatic ring, a low temperature (CH_2_Cl_2_, −50 °C) was essential to obtain the 2-acetoxydienes **40a**–**40e** with high *Z*-selectivity (*Z*/*E* = 10:1–41:1). Cyclopropenylmethyl acetate **39f** substituted by the electron-rich *p*-tolyl group effectively underwent rearrangement, but the corresponding diene **40f** decomposed rapidly. A low selectivity (*Z*:*E* = 1.8:1) was observed for the 2-acetoxydiene **40g** resulting from the rearrangement of cyclopropenylmethyl acetate **39g** substituted by an *n*-alkyl group (Scheme 18) [22].

**Scheme 19:**
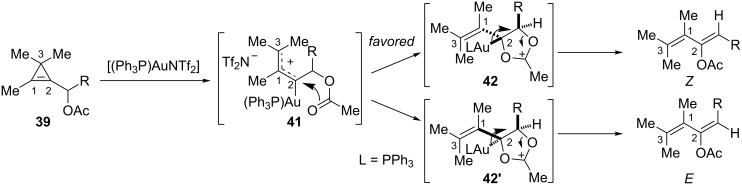
Mechanism of the gold-catalyzed rearrangement of cyclopropenes **39**.

Among the conceivable mechanisms, DFT calculations indicated that the kinetically favored pathway involved an initial regioselective electrophilic activation of the cyclopropene followed by ring-opening to yield the gold-stabilized allylic carbocation **41**. Subsequent 1,2-migration of the acetoxy group proceeded via the formation of five-membered intermediates **42** or **42'**, which then collapsed to the geometric isomers of the corresponding 2-acetoxydiene. For steric reasons, the energy barrier was found to be significantly lower for the pathway leading to the *Z* isomer, with a larger calculated difference when a phenyl group was present (R = Ph, 5.7 kcal·mol^−1^) compared to an *n*-alkyl substituent (R = Et, 1.6 kcal·mol^−1^), which correlates well with the experimental results (Scheme 19) [22].

The gold-catalyzed reactions involving cyclopropenes examined so far in this review have involved capture of the organogold intermediates, resulting from electrophilic activation and ring-opening, by an external or an internal nucleophile. Cyclopropanation of olefins, a reaction classically attributed to the carbene-like reactivity, will now be examined.

### Cyclopropanation of olefins with gold carbenes generated from cyclopropenes

#### Intermolecular cyclopropanation of olefins

**Scheme 20:**
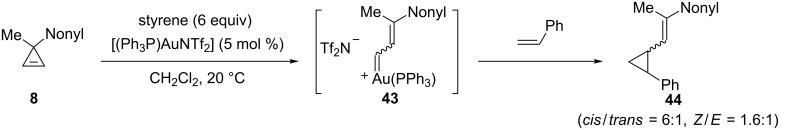
Gold-catalyzed cyclopropanation of styrene with cyclopropene **8**.

In 2008, Lee et al. disclosed several representative gold-catalyzed reactions with cyclopropenes and reported one example of cyclopropanation achieved via the gold-carbene intermediate. Thus, when 3-methyl-3-nonylcyclopropene (**8**) was treated with a catalytic amount of [(Ph_3_P)AuNTf_2_] in the presence of excess styrene, the alkenyl cyclopropane **44**, resulting from intermolecular cyclopropanation triggered by the gold carbene **43**, was isolated in 72% yield as a 6:1 mixture of *cis*/*trans* diastereomers and a 1.6:1 mixture of *Z*/*E-*geometric isomers (Scheme 20) [18].

**Scheme 21:**
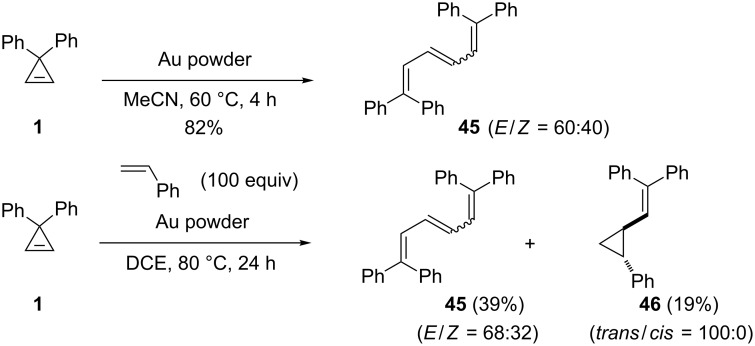
Representative reactions of carbene precursors on gold metal.

Angelici, Woo, et al. reported several catalytic reactions of carbene precursors on bulk gold metal powder consisting of particles (5–50 µm size) prepared by reduction of HAuCl_4_ with hydroquinone [23]. Upon treatment with this gold powder (MeCN, 60 °C), 3,3-diphenylcyclopropene (**1**) gave 1,1,6,6-tetraphenylhexa-1,3,5-triene (**45**), arising from self-coupling of a surface bound gold carbene, as a 40:60 mixture of *Z*/*E-*geometric isomers (82%). Cross-couplings of carbenes derived from cyclopropene **1** and phenyldiazomethane or ethyl diazoacetate on bulk gold powder were also studied, but mixtures of self- and cross-coupling products were invariably obtained with negligible selectivity. Interestingly, the authors investigated the intermolecular cyclopropanation of styrene by the surface bound gold carbene generated from cyclopropene **1**. Though a large excess of styrene (100 equiv) was used, triene **45** resulting from the self-coupling of **1** still predominated, and the cyclopropanation product **46** was isolated in low yield (19%) as a single *trans* diastereomer (Scheme 21) [23].

**Scheme 22:**
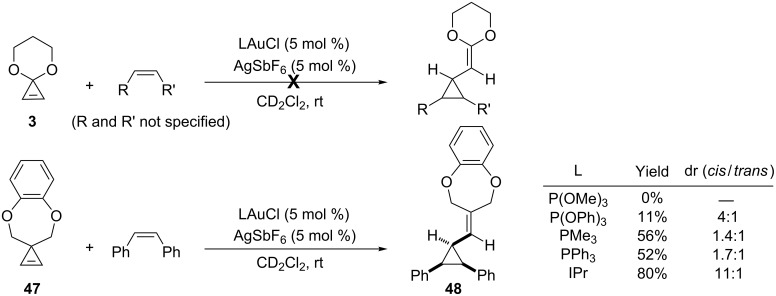
Intermolecular olefin cyclopropanation with gold carbenes generated from cyclopropenes.

In their investigations on the bonding model for gold(I) carbenoid complexes, Toste et al. highlighted the importance of the substitution pattern and the ligands (Scheme 4). Interestingly, DFT calculations were carried out for organogold species that can actually be generated by ring-opening of cyclopropenes, and therefore the authors examined experimentally the impact of cationic versus carbene-like species on the reactivity in olefin cyclopropanation [17]. In the presence of an olefin and a cationic gold(I) catalyst, cyclopropenone acetal **3** did not provide any cyclopropanation product, which is in agreement with the fact that the organogold species generated by ring-opening of **3** should instead react as a gold-stabilized carbocation due to the presence of oxygen atoms that can stabilize the cationic intermediate. However, it is worth pointing out that Boger and Brotherton previously reported that cyclopropenone acetals could cyclopropanate electron-deficient olefins, via charged intermediates, under simple thermal conditions [37]. In contrast to the behaviour of cyclopropenone acetal **3**, Toste et al. observed that the reaction of the 3,3-disubstituted cyclopropene **47** and (*Z*)-stilbene in the presence of a cationic gold catalyst could effectively provide the desired cyclopropanation product **48**, but the yield and the diastereoselectivity were highly dependent on the gold ligand. As anticipated from the structural studies, π-acidic phosphites that increase cation-like reactivity gave little or none of the cyclopropanation product **48**. Phosphines gave moderate results, whereas the highest yield and diastereoselectivity was obtained when the strong σ donor and weak π acceptor N-heterocyclic carbene IPr was the ligand. The latter was indeed anticipated to give an organogold with a higher carbene-like reactivity which favors olefin cyclopropanation. AuCl was unreactive under these conditions (Scheme 22) [17].

#### Intermolecular cyclopropanation of furans: Synthesis of conjugated trienes

**Scheme 23:**
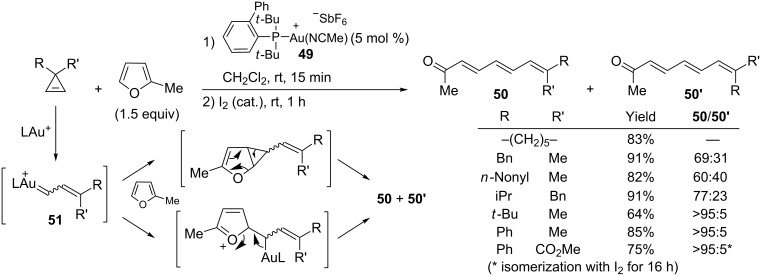
Gold-catalyzed formation of trienes from cyclopropenes and furans.

In 2011, Lee and Hadfield reported the synthesis of conjugated trienes by gold-catalyzed intermolecular reaction of cyclopropenes with furans [24]. Several catalysts such as [(Ph_3_P)AuNTf_2_], or IPrAuCl in combination with different silver salts could be used successfully, but the highest yields were obtained with the cationic gold catalyst **49**. In the presence of 2-methylfuran, a variety of 3,3-disubstituted cyclopropenes led to trienes **50**/**50’**, and the initially generated mixture of geometric isomers was isomerized by treatment with a catalytic amount of iodine. Trienes **50**/**50’** were isolated in good yields and with satisfactory levels of stereoselectivity when the steric bulk of the two substituents (R and R’) were significantly different or if a phenyl group was present. Although no cyclopropane derivative was obtained from this reaction, this transformation has been included in this section because one of the possible mechanisms involves an initial cyclopropanation of the less hindered olefin in 2-methylfuran by the organogold intermediate **51**, followed by ring-opening. The alternative mechanism involves nucleophilic attack of 2-methylfuran on the gold carbene **51**, followed by elimination (Scheme 23) [24].

**Scheme 24:**
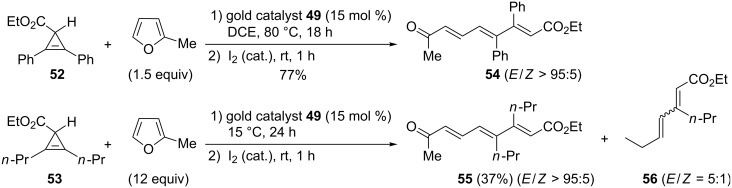
Gold-catalyzed formation of trienes from cyclopropenes and furans.

The reaction was more difficult to carry out with cyclopropene carboxylates **52** and **53** possessing tetrasubstituted alkene structures. The former substrate required harsher conditions (DCE, 80 °C), but the corresponding tetrasubstituted triene **54** was still obtained in good yield (77%). For the latter substrate, the reaction was conducted in an excess of 2-methylfuran and triene **55** was isolated in low yield (37%), accompanied by dienoate **56** as a by-product (Scheme 24) [24].

**Scheme 25:**
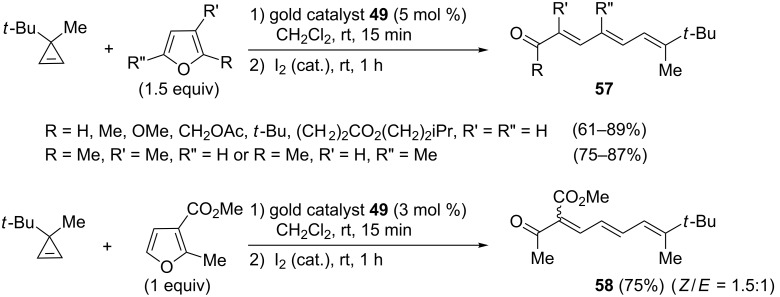
Gold-catalyzed formation of trienes from cyclopropenes and furans.

Other mono- or disubstituted furans can be successfully used as partners, as illustrated by the gold-catalyzed reactions involving 3-*tert*-butyl-3-methylcyclopropene as substrate that led to the corresponding tetra- or pentasubstituted trienes of type **57** or triene **58** bearing two geminal electron-deficient groups (Scheme 25) [24].

Besides these examples of intermolecular cyclopropanations, examples of intramolecular cyclopropanation of olefins by gold carbenes generated from cyclopropenes have been investigated in our group.

#### Intramolecular cyclopropanation: cycloisomerization of cyclopropene-enes

In 1981, Padwa et al. reported that 1,2-diphenylcyclopropenes, substituted by allyl, methallyl, crotyl groups at C3, rearranged to the corresponding 1,2-diphenylbicyclo[3.1.0]hex-2-enes upon treatment with nearly stoichiometric quantities of AgClO_4_ and prolonged heating in C_6_H_6_ or MeOH at reflux [33,38]. These reactions appear to constitute the first examples of intramolecular olefin cyclopropanation promoted by a silver carbenoid generated by ring-opening of a cyclopropene.

**Scheme 26:**
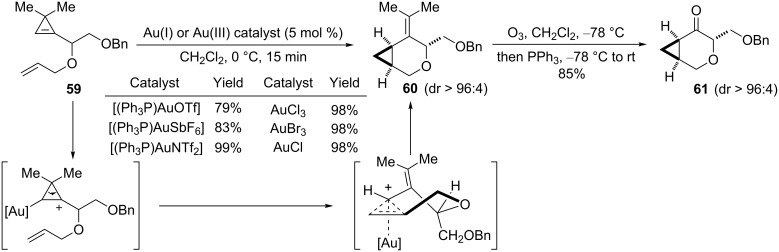
Gold-catalyzed cycloisomerization of cyclopropene-ene **59**.

We envisioned that the gold carbene resulting from the ring-opening of appropriately substituted cyclopropenes could also be involved in intramolecular olefin cyclopropanation in order to access [n.1.0] bicyclic ring systems. Rather than examining the behaviour of cyclopropenes bearing an allylic chain at C3, and in order to avoid aryl-substituted cyclopropenes that have routinely been used as substrates, allylic ethers derived from cyclopropenyl carbinols were selected as substrates. Cyclopropenyl carbinols have recently emerged as synthetically useful building blocks [39] and are readily available by the condensation of an in situ generated cyclopropenyl organolithium with an aldehyde [39–40]. Additionally, they can be obtained in an enantiomerically enriched form by Sharpless kinetic resolution [41]. In order to ensure regioselective ring-opening of the cyclopropene ring, cyclopropenyl carbinols possessing a trisubstituted endocyclic alkene were considered with the hope that a secondary cyclopropyl cation would be preferentially formed upon coordination of a gold complex. However, this implies that substituents have to be present at C3 in order to handle stable substrates. Thus, allyl 3,3-dimethylcyclopropenylcarbinyl ether **59** was prepared and several gold(I) and gold(III) species {AuCl_3_, AuBr_3_, AuCl, [(Ph_3_P)AuNTf_2_], [(Ph_3_P)AuSbF_6_] or [(Ph_3_P)AuOTf]} were found to catalyze smoothly the cycloisomerization and yield the desired oxabicyclic compound **60** in high yields and with excellent diastereoselectivity (dr > 96:4) [25]. The observed stereochemical outcome has been tentatively rationalized by considering a twist-boat transition state model in which the gold center and the allylic benzyloxymethyl substituents both occupy axial positions in order to avoid 1,3-allylic strain with the vinylic methyl groups. The isopropylidene group in compound **60** can be cleaved by ozonolysis to give the corresponding 3-oxabicyclo[4.1.0]heptanone **61** (85%), and hence, the 3,3-dimethylcyclopropene moiety appears to be an excellent surrogate of an α-diazoketone (Scheme 26) [25,42].

**Scheme 27:**
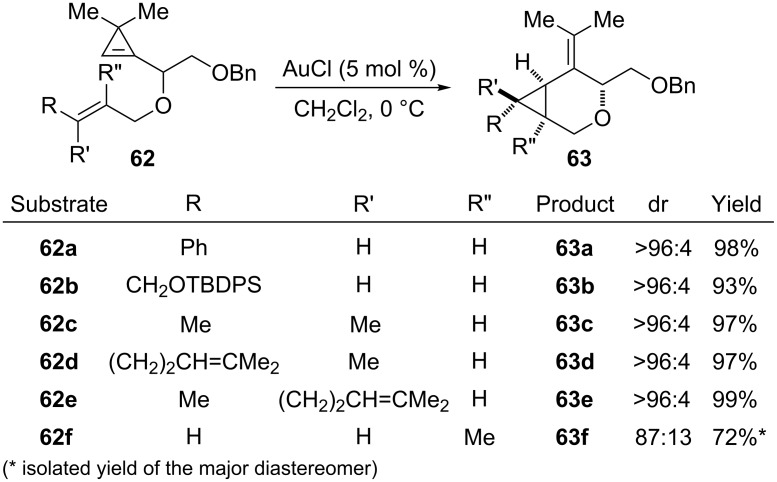
Gold-catalyzed cycloisomerization of substituted allyl cyclopropenyl carbinyl ethers **62a**–**62f**.

Further studies were carried out using AuCl as a catalyst and the reaction was generalized for a variety of substituted allylic ethers **62a**–**62f**. Excellent results were obtained with allylic ethers bearing one (**62a**, **62b**) or two substituents (**62c**–**62e**) at the terminal position of the olefin and the corresponding oxabicyclic compounds were isolated in high yields (93–99%). The stereospecificity of the cyclopropanation process was highlighted by the behaviour of geranyl ether **62d** and neryl ether **62e**, which furnished the epimeric cycloisomerization products **63d** and **63e**, respectively. The stereoselectivity was lower for methallyl ether **62f** which afforded compound **63f** as an 87:13 mixture of diastereomers (Scheme 27) [25].

**Scheme 28:**
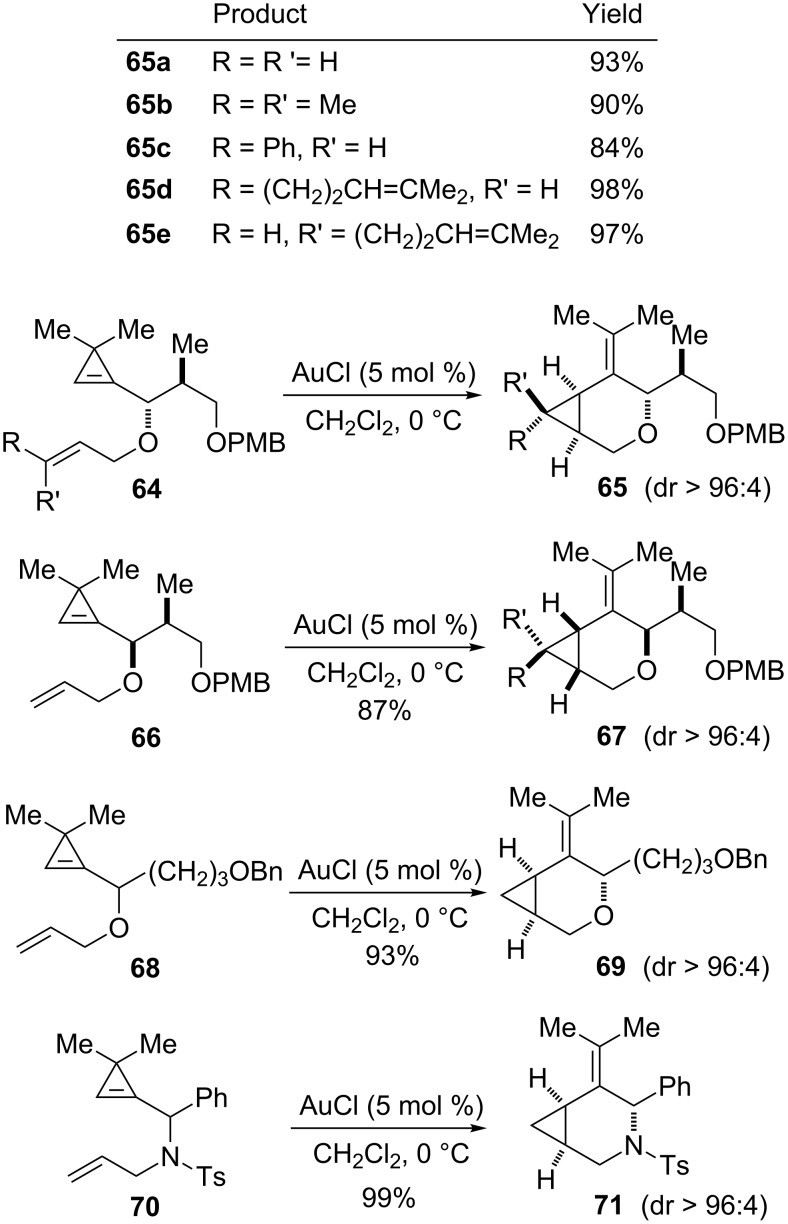
Gold-catalyzed cycloisomerization of cyclopropene-enes.

The influence of the substituent at the α-position of the oxygen atom and the cyclopropene has also been examined. Diastereoselectivities and yields were always high when this substituent was branched, whatever the relative configuration of the additional stereocenter, as shown with substrates **64a**–**64e** and **66** which led to the oxabicyclic products **65a**–**65e** and **67**, respectively. The substituent could also be a longer linear *n*-alkyl chain functionalized at the remote position by a benzyl ether, as illustrated for the cycloisomerization of **68** to **69**. Interestingly, the azabicyclic compound **71** was obtained in excellent yield (99%) and with high diastereoselectivity (dr > 96:4) by gold-catalyzed cycloisomerization of the *N*-allyl sulfonamide **70** (Scheme 28) [25].

The success of the gold-catalyzed cycloisomerization of cyclopropene-enes, proceeding with intramolecular cyclopropanation of the olefin, lies in the chemoselective activation of the cyclopropene, in preference to the alkene, which allows the generation of a gold carbene intermediate. The relative reactivity of cyclopropenes compared to alkynes is an interesting issue that has been addressed by Wang et al. during their studies on the gold-catalyzed cycloisomerization of cyclopropene-ynes [26].

### Cycloisomerization of cyclopropene-ynes

**Scheme 29:**
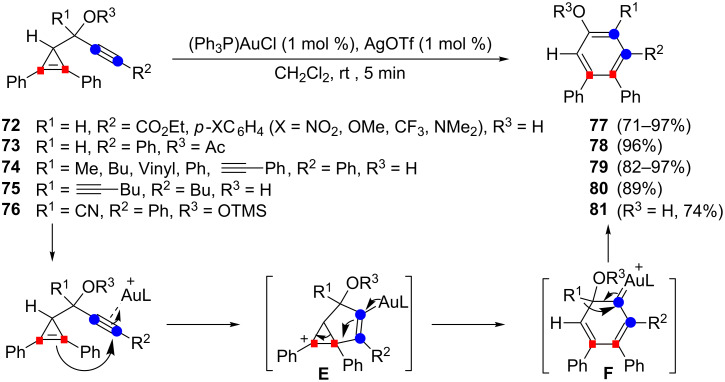
Gold-catalyzed cycloisomerization of cyclopropene-ynes.

Upon treatment with [(Ph_3_P)AuOTf] (5 mol %), several propargylic alcohols possessing a 2,3-diphenylcycloprop-2-enyl substituent were smoothly converted (CH_2_Cl_2_, rt, 5 min) to substituted 4,5-diphenylphenols. The scope of the reaction is quite broad since it could be applied to secondary propargylic alcohols **72** (or an acetate derivative **73**), to tertiary alcohols such as **74** or **75** and even to the *O*-trimethylsilyl cyanohydrin **76**. The corresponding cycloisomerization products **77**–**81** were isolated in good to excellent yields (71–97%). The hydroxyl group did not exert a particular role in this process since 1,2-diphenyl-3-propargylcyclopropene was rearranged to 1,2-diphenylbenzene (97%) under the same conditions [26]. The formation of phenols (and their derivatives) **77**–**81** could be explained by an initial chemoselective activation of the alkyne by the gold catalyst with subsequent intramolecular nucleophlic attack of the cyclopropene olefin. Ring-opening of the cyclopropyl cation **E** to generate the 1,3-cyclohexadiene **F** and a 1,2-shift of the R^1^ group would then lead to the substituted 4,5-diphenylphenol. In this mechanistic pathway, the cyclopropene carbon atoms become directly linked to those of the alkyne with no skeletal rearrangement (Scheme 29) [26].

**Scheme 30:**
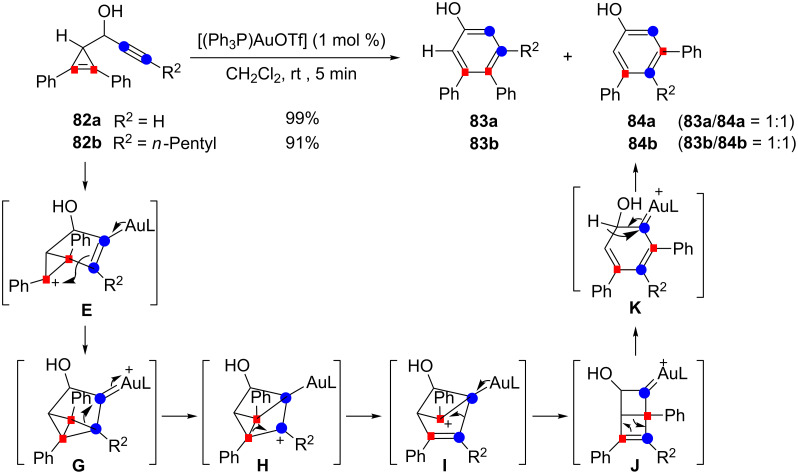
Formation of products arising from a double cleavage process in the gold-catalyzed cycloisomerization of cyclopropene-ynes.

However, the substituents were found to exert an important influence on the outcome of the reaction. Indeed, the secondary propargylic alcohols **82a** and **82b**, in which the alkyne is terminal or substituted by an *n*-pentyl group, afforded an equimolar mixture of two regioisomeric phenols (91–99%). Whereas 4,5-diphenylphenols **83a** and **83b** correspond to the previously observed rearrangement pathway, the structure of the symmetrical phenols **84a** and **84b** indicates that cleavage of both the cyclopropene double bond and the alkyne had occurred. To explain the formation of the latter double cleavage products, Wang et al. proposed a mechanistic scenario in which back donation from gold in the initially formed vinyl gold species **E** led to the highly strained gold carbene **G** possessing a tricyclo[3.1.0.0^2,6^]hexane structure. Rearrangement of **G** by consecutive 1,2-alkyl shifts, proceeding through carbocations **H** and **I** and Dewar-type benzene **J** as intermediates, followed by ring-opening and a 1,2-hydrogen shift, ultimately led to **84a** or **84b** (Scheme 30) [26].

**Scheme 31:**
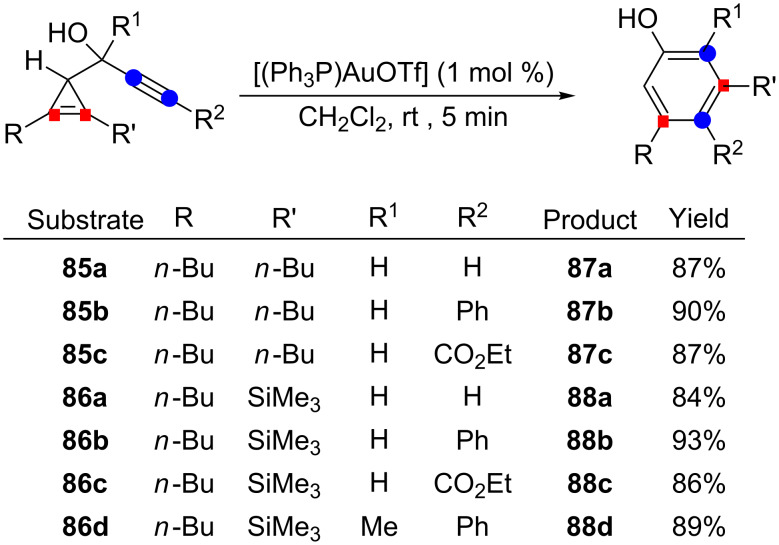
Gold-catalyzed cycloisomerization of cyclopropene-ynes involving a double cleavage process.

Since intermediates **G**–**J** are all sterically crowded, this double cleavage mechanistic pathway should be favored for cyclopropenes bearing smaller substituents. In fact, for cyclopropenes **85a**–**85c** having two *n*-butyl substituents or cyclopropenes **86a**–**86d** with one *n*-butyl and one trimethylsilyl group (the latter ensuring regioselective attack of the cyclopropene onto the activated alkyne to form a β-silylcyclopropyl cation), the gold-catalyzed rearrangement led exclusively to the phenols **87a**–**87c** and **88a**–**88d** (84–93%), respectively, resulting from a double cleavage process, whatever the substituent on the alkyne (Scheme 31) [26].

**Scheme 32:**
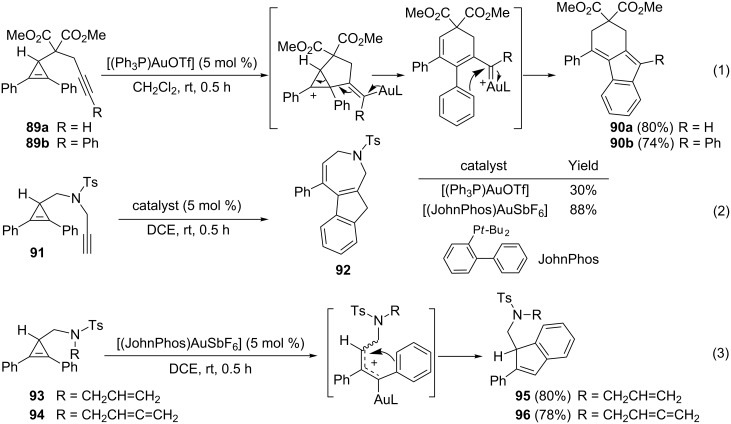
Gold-catalyzed reaction of cyclopropene-ynes, cyclopropene-enes and cyclopropene-allenes.

Wang et al. also examined the behaviour of other cyclopropen-1,n-ynes. For substrates **89a** and **89b** possessing a 1,6-enyne moiety, the gold-catalyzed cycloisomerization led to the tricyclic hydrocarbons **90a** (80%) and **90b** (74%), respectively. The alkyne, chemoselectively activated by the gold complex, underwent nucleophilic attack by the cyclopropene in a 5-*exo*-dig manner followed by ring-opening. A subsequent Friedel–Crafts cyclization allowed the formation of the indene subunit (Equation 1, Scheme 32). Sulfonamide **91** contains a 1,7-enyne subunit and its gold-catalyzed cycloisomerization delivered tricyclic compound **92** incorporating a seven-membered nitrogen heterocycle. The yield of this transformation was found to be greatly improved when in situ generated [(JohnPhos)AuSbF_6_] was used as the catalyst (88%) instead of [(Ph_3_P)AuOTf] (30%) (Equation 2, Scheme 32). When the alkyne was replaced by an alkene or an allene, the corresponding substrates **93** and **94** underwent a gold-catalyzed rearrangement to afford indenes **95** (80%) and **96** (78%), respectively. Interestingly, only the cyclopropene reacted by ring-opening followed by Friedel–Crafts cyclization: The alkene and the allene units were unaffected (Equation 3, Scheme 32) [26].

Thus, alkynes appear to be chemoselectively activated in the presence of gold complexes in preference to cyclopropenes, whereas the latter moiety is more reactive than alkenes and possibly allenes, although in the latter case only a single example of competition was reported.

## Conclusion

Though relatively recent, the entry of cyclopropenes into the area of gold catalysis has already led to interesting contributions exploiting different aspects of the reactivity of alkenyl organogold carbenoids. It is obvious that the possibility to generate gold carbenes from cyclopropenes opens new possibilities and further synthetic developments in this field will certainly be reported.
